# Gefühlt steif … und objektiv? Kann man Muskelverspannungen messen?

**DOI:** 10.1007/s00482-022-00636-y

**Published:** 2022-03-17

**Authors:** A. V. Dieterich, A. Haueise, L. Gizzi

**Affiliations:** 1 Studiengang Physiotherapie, Fakultät Gesundheit, Sicherheit, Gesellschaft, Hochschule Furtwangen, Studienzentrum Freiburg, Konrad-Goldmann-Str. 7, 79100 Freiburg i.B., Deutschland; 2 Institute for Modelling and Simulation of Biomechanical Systems, Universität Stuttgart, Pfaffenwaldring 4f, 70569 Stuttgart, Deutschland

**Keywords:** Muskelverspannung, Schmerz, Übersichtsarbeit, Scherwellenelastografie, Elektromyografie, Muscle tension, Review, Pain, Shear wave elastography, Electromyography

## Abstract

Fast jeder kennt „verspannte“ Muskulatur, aber was sind physiologisch betrachtet Muskelverspannungen? Aktivierte Muskeln, die nicht entspannen können? Steifere oder härtere Muskeln? In der vorliegenden Arbeit wird aktuelle wissenschaftliche Evidenz zur Aktivität und Steifigkeit verspannter Nackenmuskeln präsentiert und die dabei angewandten Messmethoden werden mit ihren Limitationen vorgestellt. Diese Limitationen verdeutlichen die Begrenztheit des aktuellen Stands wissenschaftlicher Erkenntnisse und den weiteren Forschungsbedarf. Abschließend wird ein aktuelles drittmittelgefördertes Forschungsprojekt zur Messung von Muskelverspannungen vorgestellt.

## Abstract (en)

Almost everyone is familiar with “tense muscles”, but what is muscle tension physiologically behind? Are tense muscles more active; do they have problems relaxing? Are they harder or stiffer than asymptomatic muscles? In this work, current evidence regarding the activity and stiffness of tense neck muscles is presented. Further, measurement methods and their limitations are explained. These limitations reveal the shortcomings of the current knowledge and the need for further research. Finally, a recently funded research project on the measurement of tense muscles is presented.

Fast jeder kennt „verspannte“ Muskulatur. Aber was sind Muskelverspannungen? Aktivierte Muskeln, die nicht entspannen können? Steifere oder härtere Muskeln? Anhand des Beispiels der schmerzhaft verspannten Nackenmuskulatur werden im Folgenden typische Annahmen, wissenschaftliche Messverfahren, daraus entstandene Erkenntnisse sowie offene Fragen und Schlussfolgerungen vorgestellt.

Chronische, vorwiegend muskuloskelettale Schmerzen betreffen weltweit ca. 31 % der Bevölkerung [38]. Ein Anteil muskuloskelettaler Schmerzen wird im Bereich der Muskulatur lokalisiert [6, 26]. Bei palpatorischer Untersuchung zeigt sich der schmerzhafte Muskel als druck- oder berührungsempfindlich. Häufig sind schmerzhafte Muskeln in der Konsistenz verändert mit allgemeinen oder lokalen Verhärtungen oder Triggerpunkten [13]. Ein zentrales Phänomen ist die „Verspannung“. Physiologisch ist unklar, was muskuläre Verspannungen sind und was sie aufrechterhält. Schmerzhafte Verspannungen im Bereich der Muskulatur begleiten regelmäßig z. B. Gelenkerkrankungen wie Arthrose oder rheumatische Arthritis. Aber am häufigsten werden Verspannungsbeschwerden vermutlich in der Nackenmuskulatur beklagt.

## Sind verspannte Nackenmuskeln zu aktiv?

Schmerzhaft oder steif empfundene Nackenmuskulatur ist ein typisches Symptom chronischer Nackenschmerzen [39], das sich oft in Zusammenhang mit länger währender monotoner Beanspruchung der Nackenmuskulatur, z. B. bei PC-Arbeit, äußert. Eine weit verbreitete Annahme ist, dass verspannte Muskeln einen höheren Aktivitätslevel, also eine zu lang andauernde elektrische Muskelerregung zeigten („können nicht lockerlassen, brauchen Entspannung“). Castelein und Kolleginnen führten eine systematische Literaturrecherche nach Studien durch, in denen die mit Elektromyografie gemessene Muskelaktivität der schulterblattführenden Muskulatur zwischen Individuen mit chronischen Nackenschmerzen und asymptomatischen Kontrollindividuen verglichen wurde [5]. In Ruhe und bei Aktivitäten bis Schulterhöhe zeigte sich kein Unterschied in der Aktivität des M. trapezius zwischen Individuen mit und ohne Schmerzen. Bei Über-Kopf-Aktivitäten waren die Ergebnisse zu heterogen, um klare Schlussfolgerungen zu ziehen [5]. Bei Zweifeln, ob kontrollierte Studienbedingungen und kurzzeitige Experimente Büroarbeitsbedingungen wiedergeben: Bereits 1996 publizierten Carlson und Kollegen eine Studie, in der zehn Frauen mit chronischen Nackenschmerzen und zehn schmerzfreie Kontrollprobandinnen drei Arbeitstage lang die Aktivität des M. trapezius jeweils über 6 h mit EMG aufnehmen ließen. Die mittlere Aktivität des M. trapezius war in der Schmerzgruppe sogar etwas niedriger, aber statistisch nicht verschieden von der Kontrollgruppe. Es gab keinen Zusammenhang zwischen Muskelaktivität und wahrgenommener Muskelspannung [4]. Kann man also die Hypothese erhöhter muskulärer Aktivität bei Nackenverspannungen verwerfen? Was genau wurde gemessen?

## Was misst Elektromyografie?

Elektromyografie (EMG) erfasst elektrische (Aktions‑)Potenziale im Muskelgewebe in Reichweite der Elektroden (= Sensoren), also die elektrische Erregung, die der mechanischen Muskelkontraktion vorausgeht. Auch geringfügige elektrophysiologische Veränderungen, z. B. bei mentalem Training [17] oder Haltungsveränderungen [8] können erfasst werden. Muskelentspannung kann durch das Fehlen elektrischer Aktivität objektiviert werden. Meistens wird das Oberflächen-EMG („surface EMG“, sEMG) verwendet. Ein Elektrodenpaar wird in Muskelfaserrichtung auf die Haut geklebt und erfasst elektrische Signale ca. 1,5–2 cm lateral und unterhalb der Elektroden [37]. Wenn mehr als ein Muskel in der Reichweite der Elektroden liegt, lässt sich nicht unterscheiden, von welchem Muskel die Signale stammen (Crosstalk). Die im Review von Castelein et al. [5] aufgeführten Studien mit sEMG haben nur die oberflächliche Muskelschicht gemessen, also den M. trapezius. Falls im Nacken gemessen wurde, könnten noch Signale des darunter liegenden M. splenius capitis erfasst worden sein.

Zur Orientierung bezüglich der Komplexität der Nackenmuskulatur folgt hier eine kurze anatomische Beschreibung. Die Oberflächenanatomie der Nackenregion wird durch den M. trapezius, insb. den Pars descendens, geprägt. Der M. trapezius ist ein Muskel des Schultergürtels. Er trägt nur geringfügig zur aktiven Nackenextension bei [10]. Darunter liegen, von oberflächlich nach tief, die autochtonen Nackenextensoren: Der M. splenius capitis verbindet diagonal die Processi spinosi des 7. Halswirbelkörpers und der ersten drei Brustwirbelkörper mit dem Processus mastoideus und der lateralen Linea nuchalis superior, er führt also eine Extension und ipsilaterale Rotation aus [41]. Darunter verbindet der longitudinal zur HWS ausgerichtete M. semispinalis capitis die Processi transversi des 3. Halswirbels bis 7. Brustwirbels mit der Squama ossis occipitalis und führt primär eine Extension des Kopfes aus [41]. Die noch tiefer liegenden Nackenextensoren inserieren nicht am Kopf. Der M. semispinalis cervicis verbindet die Processi transversi des 7. Halswirbels und der ersten sechs Brustwirbel mit den Processi spinosi des 2.–6. Halswirbelkörpers [41]. Auch der M. multifidus einschließlich der Mm. rotatores verbindet die Wirbelkörperquerfortsätze mit den Dornfortsätzen und überspringt dabei ein bis vier Segmente [41].

Will man die Aktivität tiefer liegender Muskeln messen, ist invasives EMG, z. B. mit hochflexiblen Feindrahtelektroden („fine-wire EMG“, fwEMG) erforderlich. Der haarfeine sterile Draht wird, i. d. R. unter Ultraschallkontrolle, in den Muskel injiziert. Die Injektionsnadel wird herausgezogen; der Feindraht verbleibt für die Untersuchung im Muskel und wird danach herausgezogen. Die Prozedur muss steril durch qualifiziertes Personal erfolgen und hat Tücken: Es ist schwierig, die dünne Injektionsnadel im Ultraschall zu verfolgen, also genau zu sehen, in welcher Muskelschicht der Feindraht liegt. Im Muskel selbst ist das Vorschieben der Nadel schmerzfrei, aber die Faszie ist innerviert. Wird zufällig ein Rezeptor getroffen, bleibt er gereizt, der Schmerz beeinflusst die Muskelaktivität und die Aufnahmen sind nicht verwertbar. Zudem kann die Feindrahtelektrode nur die Signale der direkt umgebenden Muskelfasern aufnehmen, also ein paar Kubikmillimeter Muskel. Sind diese Kubikmillimeter repräsentativ für den gesamten Muskel? Da für einige Muskeln eine inhomogene Aktivierung bereits dokumentiert wurde [16, 42, 44], ist die Repräsentativität eines kleinen Muskelanteils für den Gesamtmuskel unsicher. Es gibt wenige fwEMG-Studien zur Aktivität der tiefliegenden Nackenmuskulatur [35, 36]. Die bisherigen Ergebnisse weisen auf eine reduzierte und weniger zielgerichtet präzise Aktivität der tiefen Nackenmuskeln bei Nackenschmerzen hin [34]. Diese Ergebnisse werden auch durch eine funktionelle Magnetresonanzstudie unterstützt [30].

Will man exakt wissen, wo und wann Muskelanteile oder einzelne motorische Einheiten aktivieren, benutzt man hochauflösendes EMG („high-density EMG“, HDEMG). Dabei wird eine Elektrodenmatrize mit vielen dicht nebeneinanderliegenden Sensoren auf der Haut über dem Muskel fixiert. Die Analyse der zahlreichen gleichzeitigen HDEMG-Signale ist komplex und erfolgt programmiert durch Spezialisten. Für den M. trapezius weisen HDEMG-Untersuchungen bei experimentellen Nackenschmerzen auf schmerzassoziierte Veränderungen der Lokalisation und der Variabilität der Muskelaktivierung hin [15, 28].

*Die aktuelle wissenschaftliche Evidenz zeigt, dass der M. trapezius bei Nackenschmerzen nicht aktiver als bei schmerzfreien Personen ist. In Bezug auf die tiefer liegende Nackenmuskulatur existieren nur wenige Untersuchungen, welche keine aktivere tief liegende Nackenmuskulatur nachwiesen. Vermutlich liegt Nackenmuskelverspannungen keine erhöhte Muskelaktivität zugrunde. Die wissenschaftlichen Erkenntnisse unterstützen allerdings eine veränderte Verteilung der Aktivität zwischen den Nackenmuskeln *[19, 20, 35]*.*

## Sind verspannte Nackenmuskeln steifer als asymptomatische Muskeln?

Für eine Antwort müssen die mechanischen Eigenschaften des Muskelgewebes gemessen werden. Es gibt mehrere Verfahren, die entweder durch die Haut und das Subkutangewebe auf den Muskel drücken (Myoton) oder das Gewebe in Schwingung versetzen und, i. d. R. mit bildgebendem Ultraschall, Schwingungsunterschiede messen (Elastografie). Wissenschaftlich hat sich die Scherwellenelastografie durchgesetzt; nur diese Technologie ermöglicht eine lokal differenzierte und richtungsspezifische Quantifizierung von Gewebeelastizität auch für tiefer liegende Gewebe [9, 33]. Für die Messung oberflächlicher Muskeln besteht eine moderate bis gute Korrelation der Scherwellenelastografie mit Myotonmessungen [25], die aber für tief liegende Gewebeschichten nicht durchführbar sind. Das alternativ mögliche Ultraschallverfahren, die Strain-Elastografie, resultiert in qualitativen Aussagen („strain index“) zur Gewebesteifigkeit relativ zu einem Referenzwert, aber nicht in quantitativen Messwerten [33]. Bei der Scherwellenelastografie werden zusätzlich zum konventionellen bildgebenden Ultraschall mittels fokussierter Schallwellen sog. „push beams“, minimale Verschiebungen im Gewebe erzeugt. Diese Verschiebungen lösen Scherwellen aus, die sich im Gewebe fortsetzen [2]. In lockerem Gewebe leiten sich Scherwellen langsam fort, in steifem Gewebe schneller. Anhand der mit bildgebendem Ultraschall messbaren Scherwellengeschwindigkeit lässt sich die Muskelsteifigkeit, der sog. Schermodulus, errechnen [2]. Dabei entstehen farbig überlagerte Ultraschallbilder, sog. Elastogramme, die lokale Muskelsteifigkeit sichtbar machen. Im Vergleich zum sEMG bietet Scherwellenelastografie den Vorteil einer größeren Eindringtiefe in das Gewebe. Zum Bespiel können auch die tiefer liegenden Schichten der Nackenmuskulatur bis hin zum M. multifidus untersucht werden [10]. Allerdings ist die Reproduzierbarkeit der Messung in den tiefen Schichten geringer [27] und das Aufnehmen von guter Bildqualität schwerer als bei oberflächlichen Muskeln. Dennoch ist die nichtinvasive Messung tief liegender Muskulatur ein großer Vorteil gegenüber sEMG-Messungen.

Bisherige Arbeiten zum Vergleich der Steifigkeit schmerzhaft verspannter Muskeln mit asymptomatischen Muskeln zeigen mehrheitlich, aber nicht durchgängig eine erhöhte Steifigkeit bei Schmerzen. Bei temporomandibulären Beschwerden dokumentieren die Reviews von Olchowy [31] und Costa [7] drei Studien mit einer erhöhten Steifigkeit des M. masseter im Vergleich zu asymptomatischen Individuen; eine Studie zeigte keine Gruppenunterschiede [22]. In der lumbalen Rückenmuskulatur wurde bei Individuen mit Rückenschmerzen im Vergleich zu Kontrollindividuen eine höhere Muskelsteifigkeit aufgezeigt [24, 29]. Bei Migräne und Spannungskopfschmerzen wurde von einigen Autoren erhöhte Nackenmuskelsteifigkeit gemessen [1, 21], während eine weitere Studie keine Unterschiede zu schmerzfreien Individuen fand [23]. Bei Nackenschmerzen fand eine Studie für den M. trapezius, aber nicht für den M. splenius capitis, eine erhöhte Steifigkeit [40], während eine andere Studie keine statistisch signifikanten Steifigkeitsunterschiede nachwies [12]. Bereits in den 80er-Jahren des vergangenen Jahrhunderts wurde mithilfe von Resonanzmessungen an Individuen mit rheumatoider Arthritis festgestellt, dass als steif wahrgenommene Muskeln objektiv weniger steif waren als die Muskeln Gesunder [45].

Bei genauerer Betrachtung der beiden Studien zur Steifigkeit der Nackenmuskulatur [12, 40] zeigen sich diese als schlecht vergleichbar. In der Studie von Taş und Kollegen wurde mit Scherwellenelastografie die Steifigkeit des M. trapezius zwischen Akromion und C7 (also nicht im Nacken) in entspannter Bauchlage gemessen. Der M. splenius capitis wurde im Nacken gemessen. In den Elastogrammen wurden zehn kleine Messzonen innerhalb der sichtbaren Muskelfläche verteilt, ob zufällig, standardisiert oder auf ausgewählten Positionen wird nicht beschrieben. Die Messwerte in diesen zehn Messzonen (<10 % der im Elastogramm inkludierten Muskelfläche) zeigten für den M. trapezius höhere Steifigkeitswerte in der Nackenschmerzgruppe [40]. In der Studie von Dieterich und Kollegen wurden alle Schichten der Nackenmuskulatur während diverser Tätigkeiten aufgenommen. Es wurde eine automatisierte Bildanalyse durchgeführt, in der die Steifigkeit der gesamten sichtbaren Muskelfläche ausgewertet wurde, gemittelt über alle Nackenmuskelschichten und in muskelspezifischen Bildregionen. Die meisten Untersuchungssituationen (entspanntes Liegen, simulierter Bürostress, aktive Kontraktion mit ansteigender Kraft) zeigten keine Gruppenunterschiede. In der Analyse muskelspezifischer Bildregionen zeigte allein die tief liegende Muskelschicht des M. semispinalis cervicis einen statistisch signifikanten Gruppenunterschied: eine *geringere aktive Muskelspannung* in der Schmerzgruppe [12]. Beide Nackenstudien nahmen die Steifigkeit des M. splenius capitis in vergleichbarer Weise auf und kamen zum gleichen Ergebnis, nämlich keinem Gruppenunterschied. Aufgrund der unterschiedlichen Aufnahme- und Auswertungsmethodik lassen sich die weiteren Ergebnisse kaum im Vergleich diskutieren.

## Was genau wird bei Scherwellenelastografie gemessen?

Die Scherwellenelastografie wurde für die Anwendung längs in Muskelkontraktionsrichtung validiert [14]. Die longitudinale Muskelsteifigkeit wird primär durch die Spannung der Sarkomere und des die Muskelfasern umgebenden Bindegewebes bestimmt und sie korreliert mit der Muskelkraft [3]. Aber Muskeln sind in ihren mechanischen Eigenschaften in Längs- und Querrichtung, also longitudinal und transversal, verschieden (anisotrop) [18]. Die transversale Muskelsteifigkeit zeigt vermutlich die Gewebesteifigkeit durch die Verbindungen zwischen den Muskelfasern, die extrazelluläre Matrix (ECM). Man weiß, dass physiologisch auch transversal Kraft übertragen werden kann [32, 43]. Aber diese sogenannte laterale Kraftübertragung wurde bisher wenig beforscht. Klinisch wird die Konsistenz eines Muskels v. a. quer zur Muskelfaser palpiert. Massagetechniken finden großteils quer zur Muskelfaser statt. Es könnte sein, dass longitudinale Messungen von Muskelsteifigkeit limitierte Relevanz für Muskelverspannungen haben.

**Figure 1:**
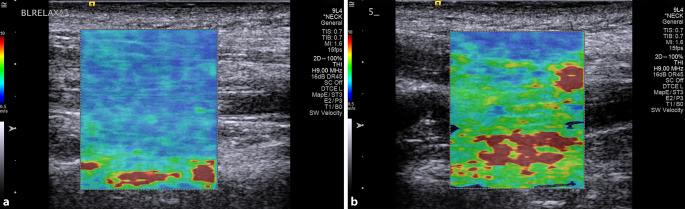

Die Scherwellenelastografie zeigt, dass die Steifigkeit in Muskeln meist inhomogen verteilt ist (Abb. 1). Zumindest bei aktiven Muskeln könnte dies überraschen, weil die anatomische Muskelfunktion als eine Muskeleigenschaft festgelegt ist und so in Sport und Therapie angenommen wird. Wie bereits erwähnt, wurde durch EMG-Messungen auch eine inhomogene Muskelaktivierung demonstriert [16, 42, 44]. Ob die inhomogene Steifigkeitsverteilung mit inhomogener Muskelrekrutierung zusammenhängt, ist bisher unklar. Auch ob die Muskelregionen erhöhter Steifigkeit etwas mit Verspannungen zu tun haben ist, unklar. In der Studie von Dieterich und Kollegen [12] konnten keine statistischen Unterschiede in der Verteilung oder Größe der steiferen Muskelbereiche zwischen den Gruppen ermittelt werden. Die inhomogene Steifigkeitsverteilung in Muskeln bedeutet für die Analyse der Elastogramme, dass die Repräsentativität kleiner Messzonen unsicher ist. Folglich sollte bei Scherwellenelastografie die gesamte im Elastogramm sichtbare Muskelfläche ausgewertet werden. Das ist in vielen Studien nicht der Fall.

Zusammenfassend sind die derzeitigen wissenschaftlichen Methoden und Erkenntnisse zur Steifigkeit der Nackenmuskulatur heterogen und lassen noch keine finalen Schlussfolgerungen zu. Hier ist weitere Forschung erforderlich.

Die Scherwellenelastografie ermöglicht neue Einblicke in die Muskelfunktion und -mechanik. Das baden-württembergische Ministerium für Wissenschaft und Kunst fördert ein zweijähriges Forschungsprojekt der Autoren, das die Veränderungen in schmerzhaft verspannten Muskeln genauer untersuchen wird. In der ersten Studie werden mit HDEMG und Scherwellenelastografie die elektrische Erregung motorischer Einheiten und die Muskelsteifigkeit während elektrischer Muskelstimulation longitudinal und transversal gemessen. Eine Hypothese ist, dass die inhomogene Steifigkeitsverteilung im aktivierenden Muskel die inhomogene elektrische Erregung (Rekrutierung motorischer Einheiten) reflektiert. Eine weitere Hypothese ist, dass nur wenig Kontraktionskraft transversal weitergeleitet wird, weil die transversalen Muskelfaserverbindungen dazu dienen, die Kontraktion benachbarter Muskelfasern zu erleichtern und Scherkräfte im Muskel zu reduzieren [11, 43]. In der zweiten Studie geht es um schmerzhaft verspannte Muskeln bei Kniearthrose. Aufgrund der zahlreichen und großflächigen HDEMG-Sensoren werden hier größere Muskeln als die Nackenmuskeln untersucht. Mit der in der ersten Studie erprobten Messmethodik sollen longitudinal und transversal Unterschiede in der Steifigkeit und der Rekrutierung schmerzhafter Muskeln untersucht werden. Dieses Forschungsprojekt strebt an, durch die Bearbeitung grundlegender Fragen die Möglichkeiten der klinischen Nutzung der Scherwellenelastografie im Bereich muskuloskelettaler Erkrankungen und Schmerzen weiterzuentwickeln und schmerzhafte Muskelverspannungen besser zu verstehen.

## Fazit für die Praxis

Physiotherapeutinnen und -therapeuten behandeln regelmäßig Muskelverspannungen. Die Therapie ist von Traditionen, Erklärungsmodellen und individuellen Erfahrungen geprägt. Wissenschaftliche Forschung durch Physiotherapeut*innen kann möglichst gezieltes und physiologisch begründetes (Be‑)Handeln ermöglichen. Durch die Akademisierung der Therapieberufe können Klinik und Wissenschaft näher zusammenrücken, mit Vorteilen für eine gute Gesundheitsversorgung.
